# Acute Transverse Myelitis (Ascending Myelitis) as the Initial Manifestation of Japanese Encephalitis: A Rare Presentation

**DOI:** 10.1155/2013/487659

**Published:** 2013-03-25

**Authors:** Varshney Ankur Nandan, Kumar Nilesh, Behera Dibyaranjan, Tiwari Ashutosh, Anand Ravi, Anand Arvind

**Affiliations:** IMS BHU, Varanasi, India

## Abstract

Japanese encephalitis, an inflammatory brain disease prevalent in Southeast Asia, usually presented with fever, headache, convulsions, brain stem signs with pyramidal and extrapyramidal features, and altered sensorium. Acute transverse myelitis, as the initial manifestation of Japanese encephalitis, is an unusual manifestation and is seldom reported. We hereby report a case of 13-year-old adolescent boy who presented to us with fever and acute onset paraparesis with urinary retention initially, progressing to quadriparesis and then followed by headache and altered sensorium. Brain MRI revealed bilateral basal ganglia that were grossly swollen with vasogenic edema tracking along internal capsule and midbrain. Adjacent ventrolateral thalamus and internal capsule also showed mild abnormal intensities. Spinal screening showed abnormal cord intensities in entire cord with gross edema in cervical and conus regions. He had elevated IgM titres against JE virus in cerebrospinal fluid. The patient was treated conservatively along with intravenous methyl prednisolone for 5 days. He regained near normal power at 3 months in followup, but hesitancy, dysarthria, and slowness of movement still persisted. To conclude, a young boy presenting with ATM in an endemic region of JE, then a possibility of Japanese encephalitis, should be sought by clinicians as early use of immunomodulator shows survival benefit.

## 1. Introduction

 Japanese encephalitis is the commonest endemic encephalitis in Southeast Asia causing significant morbidity and mortality [1]. It is caused by the JE virus belonging to family Flaviviridae, transmitted by mosquito *Culex*, and has an incubation period of 5 to 15 days. Mostly infections are asymptomatic and only 1 in 250 infections develops into encephalitis. The diagnosis is commonly based on demonstrating a rising titre of antibodies against JE virus in acute and convalescent sera [2]. The course of the disease can be divided into three stages: a prodromal stage, an encephalitic stage, and a late stage characterized by recovery or persistence of signs of CNS injury. Apart from the classical presentation other atypical presentations of JE have been reported. Among them, acute transverse myelitis had been recently reported, and this is distinctly rare. Here we describe a 13-year-old adolescent boy who presented with fever, acute transverse myelitis, and altered sensorium that was diagnosed as Japanese encephalitis.

## 2. Case Report

A 13-year-old male presented to us with fever (acute in onset, mild to moderate grade and associated with mild chills) since last 10 days followed by gradually progressive sensory motor paraparesis with urinary incontinence since last 4 days. He was then hospitalized with weakness remaining static for one day and then progressed and involved both upper limbs within 24 hours. Examination showed LMN type sensory motor quadriparesis with only flickering of contraction present in all the limbs; planter was downgoing bilaterally. The patient was febrile with other systemic and general examinations showing no significant abnormality. After one day of quadriparesis, patient developed acute onset altered sensorium preceded by drowsiness and headache which was diffuse and bursting in nature. No significant past and family history and similar episode were previous noted.

**Figure 1:**
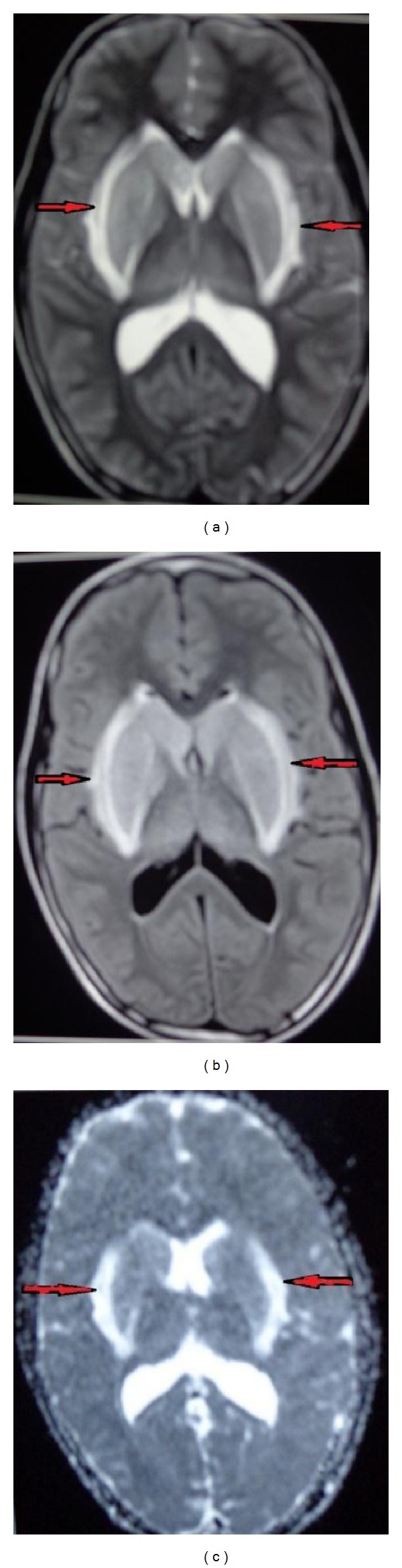
((a), (b), and (c)) Brain MRI (T1, T2, DWI) showed bilateral basal ganglia are grossly swollen with vasogenic edema tracking along internal capsule and midbrain. Adjacent ventrolateral thalamus and internal capsule also show mild abnormal intensities.

**Figure 2:**
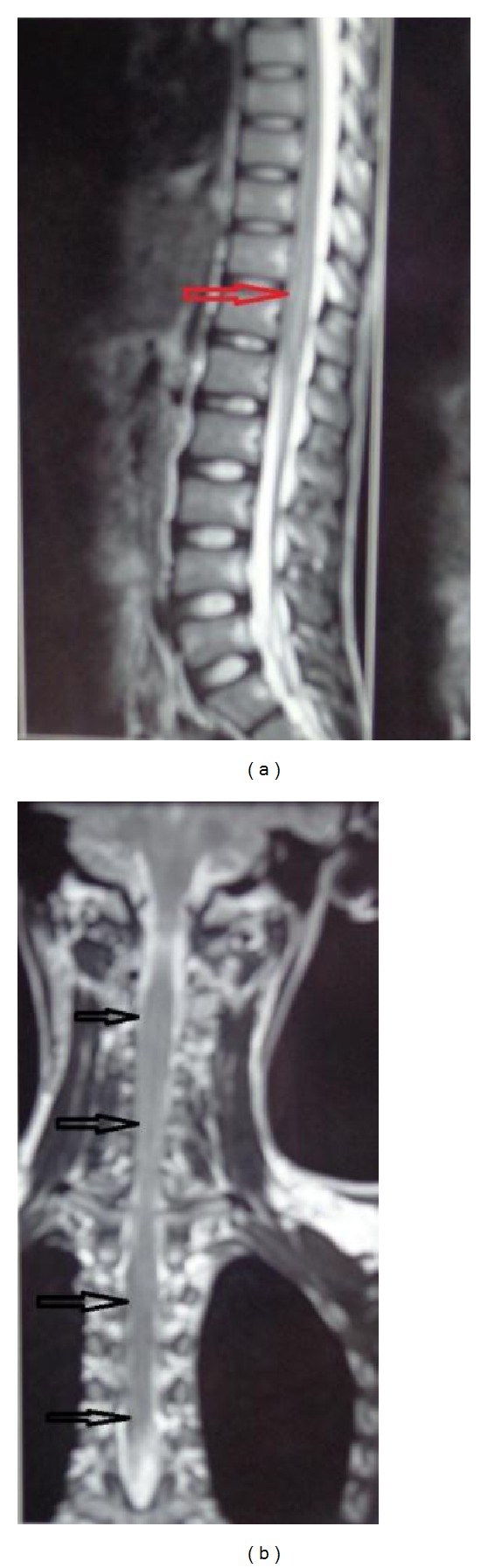
Spinal screening shows abnormal cord intensities in entire cord with gross edema in cervical and conus region.

Now examination showed a Glasgow Coma Scale of E1M1V1 and patient was deeply comatose. On the basis of this history and examination we put the diagnosis of ascending myelitis of viral origin and did investigation accordingly. As the patient belongs to an endemic region, differential diagnosis of JE was kept too. All routine investigations were within normal limit. We did CSF which revealed total leucocyte count of 42 cells/cmm with lymphocytic predominance and slight elevation of protein and normal sugar along with elevated IgM titres against JE virus. Brain MRI showed bilateral basal ganglia that were grossly swollen with vasogenic edema tracking along internal capsule and midbrain. Adjacent ventrolateral thalamus and internal capsule also show mild abnormal intensities (Figures 1(a), 1(b), and 1(c)). Spinal screening shows abnormal cord intensities in entire cord with gross edema in cervical and conus region (Figures 2(a) and 2(b)). Electrophysiological study was suggestive of preganglionic axonal involvement of upper limb and lower limb. Diagnosis of ascending myelitis and encephalitis due to JE virus was established. We started the patient on conservative treatment with intravenous methyl prednisolone for 5 days and rest treatment was symptomatic. Patient started to show improvement in the form of moving limbs and staring. Patient regained full consciousness after 2 week and was discharged and asked to be followed up in OPD. He regained near normal power at 3 months in followup but hesitancy, dysarthria, and slowness of movement persisted. This is one of the rare cases of JE which causes ascending myelitis (ATM).

## 3. Discussion

Japanese encephalitis is the leading cause of viral encephalitis in Asia, with 30,000–50,000 cases reported annually. Case-fatality rates range from 0.3% to 60% and depend on the age and on population. The natural host of the Japanese encephalitis virus is bird and many believe the virus will therefore never be completely eliminated [3]. Japanese encephalitis is diagnosed by detection of antibodies in serum and CSF by IgM capture ELISA [4]. There is no specific treatment for Japanese encephalitis and treatment is supportive, with assistance given for feeding, breathing, or seizure control as required. Raised intracranial pressure may be managed with mannitol. There is no transmission from person to person and therefore patients do not need to be isolated [5]. Neurological findings are reflections of the diffuse and asymmetric pathology in the CNS. Some degree of predilection has been described to involve the cortical gray, thalamus, hypothalamus, basal ganglia, cerebellum, and anterior horn of the spinal cord [6]. As in our case there was involvement of basal ganglia predominantly. The fine tremors in the acute and subacute periods were seen bilaterally and accentuated by movements. These tremors could not be distinguished from those due to extrapyramidal, thalamic, and cerebellar abnormalities. Choreoathetosis may also be chronic sequelae of the disease. These involuntary movements can be attributed to thalamic-subthalamic lesions and basal ganglia which are areas of predilection in JEV infections [7]. These are the commonest neurological findings in case of Japanese encephalitis.

JE may present with some atypical neurological manifestations such as involvement of various cranial nerves and changes in brain stem [8]. Papilledema and dysphagia are reported in some studies [9]. A flaccid paralysis-like illness has been reported as the initial presenting feature [10]. Hemiplegia is the presenting manifestation [11]. To the best of our knowledge, only one case of JE has been reported in which patient presented with acute transverse myelitis as an initial clinical presentation and immune-mediated cause was established [12]. Post-JE vaccination was myelitis also reported, and cellular autoimmune mechanism against the JE vaccination is suspected [13]. With early introduction of immunomodulator (as steroids), course of disease is retarded, neurological sequels are delayed and providing a survival benefit.

## 4. Conclusion

Acute transverse myelitis is one of the rare presentations of Japanese encephalitis and should be considered in patient from endemic area. Early introduction of steroid fastens recovery and diminishes neurological sequels.
